# The urinary and serum levels of IL-32 in children with febrile urinary tract infections

**DOI:** 10.4155/fsoa-2017-0076

**Published:** 2017-09-14

**Authors:** Alireza Rafiei, Hamid Mohammadjafari, Ayat Ahifar, Abbas Alipour, Araz Mohammad Mirabi

**Affiliations:** 1 Molecular & Cell Biology Research Center, Department of Immunology, Faculty of Medicine, Mazandaran University of Medical Sciences, Sari, Iran; 2 Antimicrobial Resistant Nosocomial Infection Research Center, Mazandaran University of Medical Sciences, Sari, Iran; 3 Department of Pediatrics, Faculty of Medicine, Mazandaran University of Medical Sciences, Sari, Iran; 4 Thalassemia Research Center, Mazandaran University of Medical Sciences, Sari, Iran; 5 Department of Immunology, Faculty of Medicine, Mazandaran University of Medical Sciences, Sari, Iran

**Keywords:** acute pyelonephritis, IL-32, interleukin, interleukin 32, renal scarring, vesicoureteral reflux

## Abstract

**Aim:**

We assessed the urinary and serum levels of IL-32 in pediatric patients with acute pyelonephritis (APN) with and without renal scarring.

**Results:**

We enrolled children aged 2 months to 16 years with APN. Dimercaptosuccinic acid scans and ultrasonography studies were ordered for all patients, and a voiding. A total of 86 children (aged 57 ± 39 months, 74 [86%] female) were enrolled in this study. Group 1 was composed of 19 (16 female) patients, group 2 of 38 (35 female) patients and group 3 of 29 (23 female) patients. There were no significant differences in the serum and absolute urinary levels of IL-32 (UIL-32) between groups, but the urinary IL-32/creatinine ratio (UIL-32/Cr) was significantly higher in children with pyelonephritis than controls.

## Lay abstract

Acute pyelonephritis (APN) is a serious infection for its effects on kidney structure and function. Dimercaptosuccinic acid scan is the gold standard for diagnosis of APN. The scan exposes patients to radiation and needs 6-month time to diagnosis of scar. We assessed the role of urinary and serum level of IL-32 in diagnosis of APN and anticipation of scarring. We showed that the serum and absolute urinary level of IL-32 are not helpful tools, however, urinary ratio of IL-32 to creatinine is significantly higher in pyelonephritic patients than healthy children, and could be useful. There was no difference between APN in children with scar and those without scar.

Urinary tract infection (UTI) is the second most common infection in childhood and infancy. Acute pyelonephritis (APN) is a dangerous and significant type of UTI [1]. The significance of APN is its tendency to cause permanent renal damage, also known as scarring [2,3]. Renal scarring is manifested by hypertension, chronic renal disease and severe renal dysfunction during a future pregnancy or child's life [4]. Therefore, proper diagnosis and management of APN are important standards to uphold, for both optimal treatment of a current infection and to prevent long-term complications of infection [4–6].

The diagnosis of APN is made by clinical and laboratory findings [5]. Fever with or without urinary symptoms and flank pain and tenderness are the primary clinical keys to diagnosis [1]. Urinalysis and culture with proper sampling are mandatory for definitive diagnosis [1]. There is no need to perform any additional imaging studies in most cases, but in some cases involving doubtful items in the past medical history (e.g., consumption of antibiotics before urine sampling), the use of imaging tools such as renal scans is necessary [7]. A dimercaptosuccinic acid (DMSA) scan is the most sensitive imaging tool for the diagnosis of APN [3,8].

The diagnosis of renal scarring can only be accomplished with renal imaging studies as there are no clinical findings that allow for the subjective detection of renal scarring [8]. It is important to reiterate that a DMSA scan is the most sensitive tool available to detect permanent renal damage [8].

A DMSA scan is a static renal scan that detects parenchymal inflammation in the acute phase of APN. The inflammation detected in the acute phase of the infection can resolve spontaneously or lead to permanent loss of renal parenchyma during the 4–6-month postinfection period [8].

Therefore, the diagnosis of renal scarring requires the application of an ionizing imaging tool and serving of 4–6 months’ time [8]. Recently, some other methods of diagnosing UTIs and renal scarring were studied [9]. Urinary or serum biomarkers assessed for such purposes have seen varying levels of success [10–12].

IL-32 is a pluripotent, proinflammatory cytokine with important biological functions that is expressed by natural killer cells, T cells, epithelial cells and blood monocytes [13,14]. This cytokine induces several other proinflammatory cytokines, including TNF, IL-1β, IL-6 and IL-8; therefore, it demonstrates unique proinflammatory activity. IL-32 expression increases during infection with various organisms, especially viruses [14–16]. Therefore, the cytokine could be a potentially useful marker for diagnosis, and its expression may follow any infectious or inflammatory process.

In this study, we assessed the urinary and serum levels of IL-32 in pediatric patients with APN, and compared such levels in patients with renal scarring and normal renal function.

## Materials & methods

This prospective cohort study was approved by the Ethical Research Committee at Mazandaran University of Medical Sciences and performed at Avicenna Hospital in the Department of Pediatric Nephrology, Sari, Northern Iran, between February 2014 and January 2016. All parents or relatives provided signed, written informed consent before the enrollment of their children in this study. We enrolled all children aged 2 months to 16 years with a clinical diagnosis of APN. The diagnosis of APN was suggested by a history of fever with or without urinary symptoms, flank pain or tenderness, and was confirmed by positive urine culture. A urine sample was obtained by midstream collection method in older patients, who provided appropriate cooperation for sampling. For younger children or infants in whom midstream collection could not be performed, urine collection was achieved using a catheter-based or suprapubic aspiration method. The urinalysis was considered suspicious for APN if pyuria (white blood cell count > five per high-power field), bacteriuria, positive nitrites or leukocyte esterase was reported. A positive urine culture confirmed the diagnosis, defined as a colony count of greater than 105 for midstream collection, a colony count of greater than 103 for samples collected via catheter, and any number of colonies for the suprapubic method.

Children with a history of APN, renal failure, primary or secondary immune deficiency status, and those with evidence of renal scarring on the first DMSA scan were excluded from the study.

Follow-up of patients with APN at our center was performed based on our hospital-based protocol. We performed a DMSA scan, as well as renal and urinary tract ultrasonography (US) for all patients. In an effort to detect vesicoureteral reflux (VUR), voiding cystoureterography (VCUG) was performed in patients with second episodes of APN, those with abnormal DMSA scans or US, and children with severe and atypical APN. DMSA scans were performed using a tomographic gamma camera (Siemens DH E-CAM, Wittelsbacherplatz Muenchen Germany) with a low-energy, high-resolution collimator. Inflammation was defined as an attenuation in the uptake in some or all portions of a kidney, with intact layout contour. Scarring was defined as any discontinuity in kidney contour, or any volume loss. Patients with any evidence of scarring on the initial DMSA scan were excluded from the study. DMSA scanning was repeated 4–6 months later in children with inflammatory changes.

US was performed using a Siemens G-50 scanner and 2–5 MHz curved-array transducer (Henkestr, Erlangen, Germany). For the diagnosis of VUR, a conventional VCUG was performed for male patients, and female patients underwent radionuclide cystography. The severity of VUR was classified as either mild (grade 1 or 2 on VCUG), moderate (grade 3 on VCUG) or severe (grade 4 or 5 on VCUG).

We treated patients with an appropriate antibiotic, frequently a third-generation cephalosporin or aminoglycosides, based on our local epidemiological data and bacterial sensitivity patterns.

The blood and urine samples for measuring IL-32 were obtained within the initial hours of admission. Serum was isolated from the blood sample by centrifugation at room temperature and stored at -70°C. To remove any residues or cells, all urine samples were subjected to centrifugation and then stored at -20°C for future assessment. The urinary creatinine (Cr) levels were measured using the same sample.

Serum and urine levels of IL-32 were quantified with a quantitative sandwich enzyme immunoassay using a DuoSet ELISA kit (R&D, CA, USA). Briefly, the plates were coated with goat antihuman IL-32 antibody, which served as the capture antibody, for 16 h at room temperature. Subsequently, 100 μl of standards or sera were added, and the procedure was performed according to the manufacturer's instructions. Reference concentrations of IL-32 were used to prepare the assay calibration. The absorption was determined using an ELISA reader (Biotek ELX800, Tigan St, VT, USA) at 450 nm. The concentrations were interpolated from standard curves expressed in pg/ml. Inter- and intra-assay coefficients of variation were below 10%. All samples were blindly analyzed according to clinical status to avoid any bias. All samples were run in duplicate according to the appropriate standards on Nunc MaxiSorb 96-well micro plates (Sigma-Aldrich, Schnelldorf, Germany).

Patients were divided into three groups. Patients with evidence of renal scarring on the second technetium-99m (99mTc)-DMSA scan comprised group 1. Patients with a normal first 99mTc-DMSA scan performed in the acute phase of APN and those with abnormal inflammatory 99mTc-DMSA scan findings on the first radioisotope scan but who had completely normal findings on the later scan, were both enrolled in group 2. We enrolled a group of healthy children without any previous history of renal disorders and normal antenatal renal sonography who also had normal urinalyses and culture studies as the control group. The group 3 (control group) patients were selected from children who came to our hospital for routine pediatric care.

The continuous variables showed non-normal distributions (assessed with the Kolmogorov–Smirnov test) and were expressed as mean ± standard deviation. We used the Mann–Whitney U test to compare numerical data regarding the partial distribution of the two groups, and we used the Kruskal–Wallis test for comparisons among multiple groups. The best cut-off values for serum and urine IL-32 and the urinary IL (UIL)-32/Cr ratio was determined by receiver operating characteristic (ROC) analysis and area under the curve calculations. Categorical variables were analyzed with a χ^2^ test and expressed as numbers (percentages). A p-value <0.05 was considered statistically significant. All statistical analyses were performed using SPSS software, version 22.0 (SPSS, Inc., IL, USA), except for the cut-points estimation and accuracy of those points, which were analyzed using the R programming environment (optimal cut points package).

## Results

A total of 86 children were enrolled in this study. The mean patient age was 57 ± 39 months, and 74 of the patients (86%) were female. The patients were categorized into three groups. Group 1 was composed of 19 (16 female) patients, group 2 of 38 (35 female) patients and group 3 of 29 (23 female) patients. Study demographics and other patient characteristics are summarized in Table 1. No significant differences in sex and age were observed between the groups (p = 0.315 and p = 0.27, respectively). VCUG was performed on 42 out of 57 patients with APN. Of these 42 patients, 19 (45%) had VUR, and the frequency of VUR was 74% (14 of the 19 VCUGs performed) in group 1, and 22% (5 of 23) in group 2. The severity of VUR in both groups is presented in Table 1. As expected, the frequency of VUR was significantly higher in group 1 than in group 2 (p = 0.001).

**Table 1.** **Some demographic and baseline data of three group of children.**

**Characteristics**	**Groups**			**p-value**

	**Group 1 (n = 19)**	**Group 2 (n = 38)**	**Group 3 (n = 29)**	
Age, months, mean ± SD	63 ± 37	59 ± 47	39 ± 22	0.27

Sex, F/M	16/3	35/3	23/6	0.32

VUR, n (%)	14 (74)	5 (22)	–	0.002

VUR severity, n (%):				

– Mild	4 (28.6)	2 (0.4)	–	0.007

– Moderate	4 (28.6)	2 (0.4)	–	–

– Severe	6 (42.8)	1 (0.2)	–	–

APN: Acute pyelonephritis; VUR: Vesicoureteral reflux.

## Biomarker measurement

The mean levels of urinary and serum levels of IL-32 and UIL-32/Cr ratios are presented in Table 2. As shown in Table 2, there were no significant differences in serum and absolute urinary levels of IL-32 between the groups (p > 0.05). The IL-32/Cr ratio was significantly different between the 3 groups. This ratio was higher in group 1, and higher in group 2 than in group 3. These differences were statistically significant (p = 0.04 and p = 0.02, respectively), but the difference between group 1 and 2 was not statistically significant (p = 0.83).

**Table 2.** **Urine and serum concentration of IL-32 and UIL-32/Cr in three groups of children.**

**Variables measured**	**Group**			**p-value**	**Statistically significancy between group**

	**Group 1 (n = 19)^†^**	**Group 2 (n = 38)^†^**	**Group 3 (n = 29)^†^**		
Urine IL-32 (pg/ml)	49.92 ± 17.6	55.95 ± 35.83	44.54 ± 10.21	0.58	–

Urine IL-32/Cr (pg/mg)	1.94 ± 1.20	2.36 ± 2.30	1.31 ± 0.95	0.049	A vs C-B vs C-AB vs C

Serum IL-32 (pg/ml)	1779 ± 2285	969 ± 1520	1251 ± 1563	0.47	–

^†^Mean ± SD

APN: Acute pyelonephritis; Cr: Creatinine.

Additionally, we assessed the levels of biomarkers in 19 patients with VUR and 23 patients without VUR. The mean IL-32/Cr ratio was not significantly different between the children with VUR and those with normal VCUGs (2.08 ± 1.93 pg/mg vs 2.06 ± 1.57 pg/mg, p = 0.745).

## Receiver operating characteristic analysis

**Figure 1.**
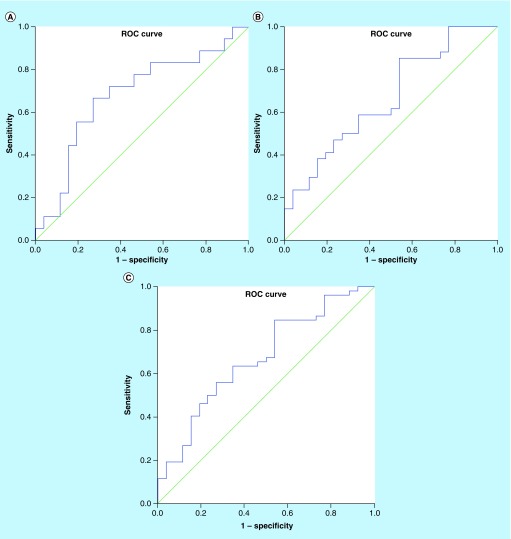
The receiver operating characteristic curve of urine IL-32 to creatinine for distinguishing **(A)** group 1 (Area under curve and scar) vs group 3 (Controls); **(B)** group 2 (Area under curve without scar) vs group 3 and **(C)** groups 1 + 2 vs group 3. ROC: Receiver operating characteristic.

The sensitivity, specificity, positive predictive value and negative predictive value of the UIL-32/Cr ratio were calculated from ROC curves (Figure 1) in order to assess the role of the test in the diagnosis of renal scarring, using comparisons between groups 1 and 2. The value was also calculated to compare its values in group 3 with the two other groups to assess the role of the UIL-32/Cr ratio in the diagnosis of APN. The cut-off values presented in each case are shown in Table 3. As shown in this table, the optimal cut-off value of the IL-32/Cr ratio for the diagnosis of APN with renal scarring was 1.42 pg/mg (AUC = 0.68; 95% CI: 0.51–0.85). The optimal cut-off value of the IL-32/Cr ratio for the diagnosis of APN without renal scarring was 0.86 pg/mg (AUC = 0.67; 95% CI: 0.53–0.81). The optimal cut-off value of the IL-32/Cr ratio for the diagnosis of APN was 0.86 pg/mg (AUC = 0.67; 95% CI: 0.54–0.8).

**Table 3.** **Cut-off value of urinary IL-32 to creatinine ratio for comparison of three group of children.**

**Groups were compared**	**Cut-off value**	**Sens**	**Spec**	**PPV**	**NPV**	**AUC**	**Standard error**	**95% CI**
Group 1 vs 3	1.40	67	73	63	76	0.682	0.085	0.516–0.848

Group 2 vs 3	1.27	59	65	69	55	0.665	0.071	0.527–0.803

Group 1 and 2 vs 3	1.27	64	65	79	53	0.671	0.065	0.544–0.798

NPV: Negative predictive value; PPV: Positive predictive value; Sens: Sensitivity; Spec: Specificity.

## Discussion

APN is considered a serious infection, both due to its high risk of acute morbidity and late sequelae affecting renal structure and function. We assessed the role of urinary and serum levels of IL-32 in the acute diagnosis of APN, and anticipation of renal scar formation potency. We showed that the serum and absolute urinary levels of IL-32 were similar between patients with APN (with and without scarring) and healthy children. In our study, the UIL-32/Cr ratio was significantly higher in patients with pyelonephritis than in healthy children, but there was no significant difference between the ratio in children with APN and renal scarring and those with APN without scarring.

Our study is a novel and unique work; we searched the literature for similar articles involving the topic of IL-32 associated with UTI and found no such studies. IL-32 has been assessed in association with some other diseases, including allergic rhinitis [17], rhinosinusitis [18], lupus erythematosus and other autoimmune disorders [19–21].

IL-32 can regulate or be regulated by several cytokines, including IFN-λ1, IL-6 and IL-8. This function makes IL-32 a crucial mediator of the host's defense response [14].

The role of other interleukins in APN has been presented in recent studies. Jantausch *et al*. assessed UIL-6 levels in a study population that consisted of 37 patients with febrile UTI and compared it with 37 febrile control children who were age-, race- and sex-matched to the patient population. She also measured the UIL-8 concentrations for a subset of 32 patients with APN and their matched controls. Median UIL-6 concentrations at the time of admission were significantly higher in the patients than in the controls (397 pg/ml compared with 0 pg/ml) (p < 0.0001). Median UIL-8 concentrations at the time of admission were 5809 pg/ml in the 32 patients compared with 0 pg/ml in the 32 controls (p < 0.0001) [22]. Sheu *et al*. measured the serum and urinary levels of IL-6 and IL-8 in 78 patients experiencing their first UTI (42 with APN, 36 with cystitis) and 12 healthy children. They found that the serum and urinary levels of IL-6 and IL-8 were significantly higher in patients with APN than in those with cystitis and no UTI [23]. Renata *et al*. studied two groups consisting of 31 children with a first episode of febrile UTI and a control group of 22 children with fever unrelated to UTI. Median UIL-6 concentrations at the time of diagnosis were 74.46 pg/ml in the study group compared with 10.51 pg/ml in the control group (p = 0.0001). UIL-6 levels had a sensitivity of 96.7% and a specificity of 18.18% in detecting APN. UIL-8 was not detected in the urine of the control group, indicating a specificity of 100% [24]. In our study, we found a sensitivity and specificity of up to 85 and 73%, respectively, which are relatively lower values compared with those of Renata's study. Key differences between the studies and ours are the different assessment methods applied; we used a more quantitative method than they used. Furthermore, Krzemień *et al*. assessed a total of 35 children in three groups: patients with APN, nonfebrile UTI and asymptomatic bacteriuria, and found that urine concentrations of IL-6 and IL-8 were significantly higher in patients with APN compared with those of the other two groups (p < 0.5, p < 0.01) [25].

In another study, Sheu *et al*. showed the initial serum and urine IL-8 concentrations were significantly higher in children with APN than in those with a lower UTI and the healthy controls [26].

We measured the levels of IL-32 in the serum and urine of children with APN. As an inflammatory marker, the urine levels were significantly higher in the patients than in healthy children. The proinflammatory role of IL-32 in viral and some bacterial infections was confirmed recently, but no published data were found about its role in UTI. The diagnosis of UTI is primarily a clinical assessment; there is no need to perform laboratory tests, except for urinalysis and culture, to diagnose APN. In some unusual or atypical cases, such as partially treated UTIs, it is necessary to confirm APN using other diagnostic tests such as a DMSA scan. The UIL-32/Cr ratio might be an alternative way to confirm UTI in such doubtful cases, but such a conclusion requires the performance of additional larger multicenter studies. Our study demonstrated relatively good sensitivity and specificity of the UIL-32/Cr ratio in diagnosing APN.

Additional significant data presented in our study included the accuracy of cytokines in detecting the potential of renal scar formation. We found no difference between the two groups of children with pyelonephritis, those with and without subsequent renal scarring. Sheu *et al*. studied 41 children with APN and 34 patients with lower UTI. They measured the urinary levels of IL-1ß and found that the level of the cytokine and the IL-1ß/Cr ratio were significantly higher in patients with APN than in those with lower UTI [27]. Tramma *et al*. evaluated IL-6 and IL-8 concentrations in the urine of 50 children in two groups: children with renal scars and children with a history of APN without renal scars. UIL-8 levels were below the lower detection limit in all samples. IL-6 was detectable in the majority of children with renal scarring, and was below the detection limits in the urine samples of children without scarring. There was a significant relationship between the grades of renal scars (p < 0.0001) [28]. In another study, Sheu *et al*. measured the level of IL-6 in the urine and serum of 45 children with APN and 34 children with cystitis. They found that the higher serum and urine IL-6 levels were associated with renal scarring in children with APN. The sensitivity and specificity used to distinguish children with and without renal scarring were 33.3 and 93.1%, respectively, for serum IL-6, and 46.7 and 89.7%, respectively, for urine IL-6 [29].

Additionally, IL-8 was assessed in a recent study. The serum and urinary levels of IL-8 were significantly higher in children with renal scarring than in those with normal DMSA scans. The ROC curves showed that the sensitivity and specificity used to distinguish children with and without renal scarring were 65.4 and 92.7%, respectively, for serum IL-8, and 76.9 and 90.2%, respectively, for urine IL-8 [26].

In our study, the initial levels of serum and urinary IL-32 were not different in the children with pyelonephritis that demonstrated a tendency to develop permanent renal damage. This means that IL-32 may be a reliable tool for the diagnosis of APN, but not for the anticipation of renal scar formation. Furthermore, Galanakis *et al*. reported that no significant difference was found between patients with renal damage and healthy patients [30].

Notably, VUR was an important variable in our study. The group 1 patients had a significantly higher incidence of VUR than that of group 2. The serum and urinary levels of IL-32 were similar in those with and without VUR; they were also similar in those with and without renal scarring.

Many years ago, Haraoka *et al*. evaluated IL-6 and IL-8 levels in the urine of 32 children with VUR. He found there were statistically significant differences between UIL-8 levels in children with and without renal scarring, and in those with and without VUR [31]. Furthermore, Mahyar *et al*. divided 80 children experiencing their first febrile UTI into two groups, those with and without VUR, based on the results of VCUG. There was neither a significant difference between the children with and without VUR, nor between low- and high-grade VUR groups regarding serum concentration of IL-8 (p > 0.05). Based on an ROC curve, the sensitivity, specificity, likelihood ratio and accuracy of serum IL-8 were lower than those of erythrocyte sedimentation rate and C-reactive protein [32]. Similarly, the serum level of IL-32 was similar between the three groups of patients in our study.

Galanakis *et al*. evaluated urine concentrations of IL-8 in 59 infants aged 1 month to 2 years. The patients were divided into three groups: group A, subjects with proven VUR; group B, subjects with a history of UTI but negative for VUR; and group C, subjects without any history of an acute or chronic condition that might impair renal function. The UIL-8/Cr ratio concentrations were significantly higher in group A than in groups B and C, but no significant difference in the measurement was observed between groups B and C. In group A, no significant correlation was shown between the UIL-8/Cr ratio concentrations and reflux grade. The sensitivity of this marker in diagnosing VUR was 88%, the specificity 69%, the positive predictive value 66% and the negative predictive value 89% [30]. Tramma *et al*. reported that there were no statistically significant differences between UIL-6 levels in children with and without VUR [28].

In two different studies, Sheu *et al*. reported the relationship between IL-6 and IL-8 in patients with VUR. They showed that there was no significant difference between urinary IL-6, IL-6/Cr ratio, IL-8 and IL-8/Cr ratio in patients with and without VUR [23,26].

## Conclusion

In conclusion, our study revealed that serum and absolute urinary levels of IL-32 are not helpful tools in diagnosing APN, renal scarring or VUR. The UIL-32/Cr ratio is not a useful measurement for the anticipation of renal scarring or VUR, but it is helpful in confirming the diagnosis of APN.

## Future perspective

APN is a serious infection in the field of pediatric nephrology both in terms of acute morbidity and late sequelae on kidney structure and function. The diagnosis can be made only with radiating measures. Our study showed that the urinary ratio of IL-32 to Cr was significantly higher in pyelonephritic patients than healthy children, but there was no significant difference between APN children with scar and those without scar. We assessed a relatively small group of children. The assessment of IL-32 and other markers could help us to find newer tools without the need for any radiation exposure for diagnosis of APN and renal scarring. These newer tools could also give us a time saving: DMSA need at least 4–6 months’ time to detect scar formation. Is it possible to anticipate the potency of scar formation from first days of APN? Future studies will help answer to this question.

**Summary points**

- The urinary and serum levels of IL-32 in pediatric patients with acute pyelonephritis (APN) were assessed.
- These levels were compared in patients with and without renal scarring, as well as those with healthy renal function.

**Methods**

- Children aged 2 months to 16 years with APN were enrolled.
- Dimercaptosuccinic acid (DMSA) scans and ultrasonography studies were ordered for all patients, and a voiding cystoureterography was ordered for children with an abnormal ultrasonography or DMSA scan.
- The serum and urinary concentrations of IL-32 and the urinary level of creatinine (Cr) were measured.
- Patients were divided into three groups: patients with APN and renal scarring were designated as group 1, patients with APN and normal scans as group 2 and healthy/control patients as group 3.

**Results**

- A total of 86 children (aged 57 ± 39 months, 74 [86%] female) were enrolled in this study. Group 1 was composed of 19 (16 female) patients, group 2 of 38 (35 female) patients and group 3 of 29 (23 female) patients.
- There were no significant differences in the serum and absolute urinary levels of IL-32 between groups.
- The urinary IL (UIL)-32/Cr ratio was significantly higher in groups 1 and 2 compared with that of group 3 (1.94 ± 1.20 pg/mg, 2.36 ± 2.30 pg/mg and 1.31 ± 0.95 pg/mg for groups 1, 2 and 3, respectively) [p = 0.04 and p = 0.02, respectively].
- The difference between the UIL-32/Cr ratio in groups 1 and 2 was not statistically significant.
- The sensitivity and specificity of the UIL-32/Cr ratio for the diagnosis of APN were 67–85% and 46–73%, respectively.

**Conclusion**

- The serum and absolute urinary levels of IL-32 were similar between children with APN (with and without renal scarring) and healthy children.
- The UIL-32/Cr ratio was significantly higher in children with pyelonephritis than in healthy children, but was similar in children with both types of APN (i.e., those with and without renal scarring).
